# Components of the antigen processing and presentation pathway revealed by gene expression microarray analysis following B cell antigen receptor (BCR) stimulation

**DOI:** 10.1186/1471-2105-7-237

**Published:** 2006-05-02

**Authors:** Jamie A Lee, Robert S Sinkovits, Dennis Mock, Eva L Rab, Jennifer Cai, Peng Yang, Brian Saunders, Robert C Hsueh, Sangdun Choi, Shankar Subramaniam, Richard H Scheuermann, in collaboration with the Alliance for Cellular Signaling

**Affiliations:** 1 Department of Pathology, Laboratory of Molecular Pathology, University of Texas Southwestern Medical Center, Dallas, Texas 75390, USA; 2 Department of Pharmacology, University of Texas Southwestern Medical Center, Dallas, Texas 75390, USA; 3 San Diego Supercomputer Center, University of California, San Diego, California 92122, USA; 4 Department of Bioengineering, University of California, San Diego, California 92122, USA; 5 Division of Biology, California Institute of Technology, Pasadena, CA, USA

## Abstract

**Background:**

Activation of naïve B lymphocytes by extracellular ligands, e.g. antigen, lipopolysaccharide (LPS) and CD40 ligand, induces a combination of common and ligand-specific phenotypic changes through complex signal transduction pathways. For example, although all three of these ligands induce proliferation, only stimulation through the B cell antigen receptor (BCR) induces apoptosis in resting splenic B cells. In order to define the common and unique biological responses to ligand stimulation, we compared the gene expression changes induced in normal primary B cells by a panel of ligands using cDNA microarrays and a statistical approach, CLASSIFI (*Cl*uster *Assi*gnment *f*or Biological *I*nference), which identifies significant co-clustering of genes with similar Gene Ontology™ annotation.

**Results:**

CLASSIFI analysis revealed an overrepresentation of genes involved in ion and vesicle transport, including multiple components of the proton pump, in the BCR-specific gene cluster, suggesting that activation of antigen processing and presentation pathways is a major biological response to antigen receptor stimulation. Proton pump components that were not included in the initial microarray data set were also upregulated in response to BCR stimulation in follow up experiments. MHC Class II expression was found to be maintained specifically in response to BCR stimulation. Furthermore, ligand-specific internalization of the BCR, a first step in B cell antigen processing and presentation, was demonstrated.

**Conclusion:**

These observations provide experimental validation of the computational approach implemented in CLASSIFI, demonstrating that CLASSIFI-based gene expression cluster analysis is an effective data mining tool to identify biological processes that correlate with the experimental conditional variables. Furthermore, this analysis has identified at least thirty-eight candidate components of the B cell antigen processing and presentation pathway and sets the stage for future studies focused on a better understanding of the components involved in and unique to B cell antigen processing and presentation.

## Background

Naïve mature B cells in peripheral lymphoid organs respond to a variety of extracellular signals through the activation of signal transduction pathways initiated by the B cell antigen, pattern-recognition, cytokine and chemokine receptors. B cell responses to signaling depend on the combination of ligands present, and include activation, proliferation, migration, differentiation, isotype class switching, somatic hypermutation, anergy, and apoptosis [1,2]. Once activated, B cells can also serve as antigen presenting cells that preferentially present antigens recognized by their specific BCR. In contrast, dendritic cells and macrophages present varied antigens that are acquired less specifically through phagocytosis, macropinocytosis and receptor-mediated endocytosis via pattern-recognition receptors such as the mannose receptor.

BCR-specific antigen processing and presentation is initiated by BCR-mediated signal transduction triggered by antigenic stimulation [3,4]. Antigen is then internalized by receptor-mediated endocytosis and trafficked through endosomes for acidification and fusion with lysosomes containing pH-sensitive hydrolytic enzymes for antigen processing. Endolysosomes containing processed antigenic peptides fuse with Golgi-derived vesicles containing MHC class II molecules assembled with invariant chain (Ii). The CLIP fragment of Ii bound in the cleft of the class II aβ dimer is replaced by antigen-derived peptides and the complex trafficked to the cell surface through vesicle secretory pathways.

It is well known that B cell antigen processing and presentation mediated through the BCR far exceeds the efficiency of presentation of the same antigen by macrophages or dendritic cells [5]. The mechanism giving rise to this increased efficiency has not been fully determined but appears to be a unique aspect of BCR-mediated antigen capture and processing as opposed to changes in the basic antigen processing and presentation machinery [6]. One mechanism that may contribute to efficiency is accelerated trafficking of BCR/antigen complexes to Class II containing vesicles inside the cell [7]. However, the molecular mediators of this vesicle trafficking, especially those components uniquely involved in the highly efficient B cell antigen processing and presentation pathway, have remained largely unknown.

We analyzed a B cell microarray dataset comparing the responses of normal splenic B cells to 32 individual ligands. This study was designed to determine functionally important input signals to contribute to the understanding of normal B cell biology and differs from existing B cell microarray studies which largely compare normal B cells to B cell lymphomas to identify tumor-specific gene expression changes. By combining this gene expression microarray analysis with bioinformatics analyses and functional assays, we have identified a set of specific genes that appear to be involved in BCR-mediated antigen capture, vesicle function and vesicle trafficking during B cell antigen processing and presentation. These data provide a foundation for the molecular characterization of this important immunological process.

## Results

### Microarray analysis of ligand-treated B lymphocytes

**Figure 1:**
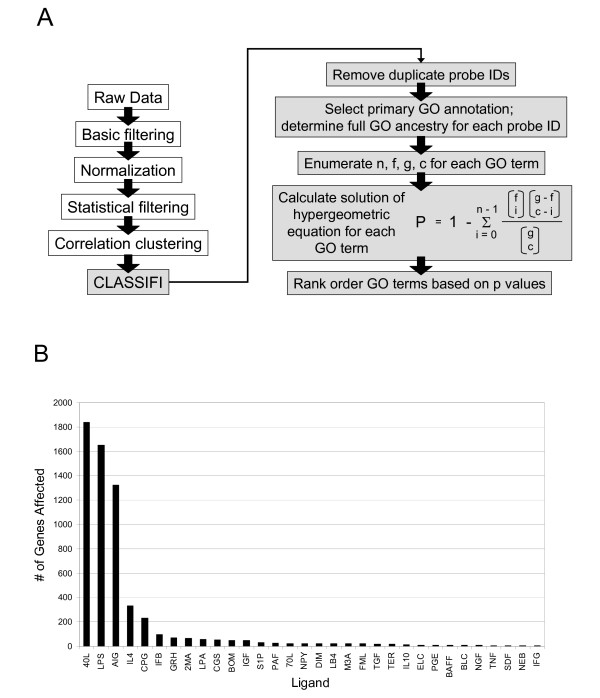
**Experimental methodology and analysis of microarray data**. (A) Schematic of steps performed during microarray data processing. Gray boxes indicate steps performed as part of the CLASSIFI component of the analysis. See text for details. (B) The number of genes whose expression was significantly changed at the 4 hr timepoint as determined by SAM is shown for each of 32 ligands. Abbreviations and descriptions for each ligand can be found at [22]. 40L = CD40L (anti-CD40), LPS = Lipopolysaccharide, and AIG = anti-IgM.

Purified B lymphocytes were treated in culture with 32 different ligands over a timecourse of 30 min, 1 hr, 2 hr, and 4 hr. A detailed description of the data set has been published [8]. Following filtering, normalization and SAM analysis, genes that were differentially expressed by each ligand in comparison with time-matched, untreated controls were identified. Of the 32 ligands, CD40L, LPS, and AIG caused the most gene expression changes, especially at the 4 hr timepoint (Figure 1B). Further analysis focuses on these three immunologically-important ligands. Categorical values of 1, -1, and 0 (representing significantly upregulated, downregulated, or unchanged) were used to group genes together based on their expression response patterns (Table 1). Genes not differentially expressed under at least one treatment condition were omitted from further analysis. Based on 3 ligand combinations and a possibility of 3 outcomes for each ligand (1, -1, 0), there are a possible 3^3 ^or 27 gene clusters, with the (0, 0, 0) outcome excluded since only genes that were differentially expressed under at least one treatment condition were selected (26 possible gene clusters). In our dataset, we only observe 19 of these possible 26. A variety of different expression patterns were observed. For example, Gene Cluster #1 contains genes that are upregulated by all three ligands, whereas Gene Cluster #14 contains genes that are only upregulated in response to LPS.

### CLASSIFI analysis links gene clusters to cellular physiology

Microarray expression data can be full of experimental and biological noise, and many microarray probes are not well characterized. We developed a gene cluster classification method that circumvents these limitations and links biological function with gene expression patterns derived from microarray experiments. This method, termed CLASSIFI, uses Gene Ontology™ annotation to identify significant co-clustering of genes with similar biological properties, based on the postulate that genes involved in the same biological process would be coordinately expressed.

CLASSIFI utilizes the gene description database developed by the Gene Ontology™ (GO) Consortium [9] to reveal co-clustering of genes with similar biological properties. The GO annotates gene products with GO terms based upon their molecular function, biological process, and cellular component. These descriptive terms are structured in a directed acyclic graph of parent-child relationships, allowing a term to have multiple parents [10,11]. GO gene annotation has also been used by other groups as a tool for identifying biological processes relevant to gene expression profiling experiments [11-13].

CLASSIFI measures the probability of co-clustering for every gene ontology term designated to every gene in each gene cluster. Gene expression data clustering and gene cluster membership assignment generates the CLASSIFI input file (See Additional file 1). CLASSIFI captures all of the GO terms annotated to every probe and then calculates the probability of chance co-clustering of GO terms. The final CLASSIFI output is a list of every GO term, ranked within each gene cluster from lowest to highest probability (See Additional file 2).

The filtered data set contained 2545 probes, which led to the calculation of 5036 probabilities (See Additional file 3). The GO term with the lowest probability for each gene cluster is shown in Table 1. A significance cutoff of 1 × 10^-5 ^was established using a Bonferroni correction with an *alpha *of 0.05 and an *n *of 5036 (see [14] and references therein). Six gene clusters give rise to GO terms with significant probabilities: Gene Cluster #1, 6, 7, 8, 14, and 18. Gene clusters identified by CLASSIFI to give significant probabilities exhibit predictable expression patterns that could be explained biologically, including gene clusters characterized by ligand-specific expression induction. Experimental noise may contribute to gene clusters with insignificant probabilities that show unusual expression patterns.

CLASSIFI results for some gene clusters are expected based on our current understanding of B cell physiology. For example, the GO term giving the lowest probability in Gene Cluster #1 is "nucleus". This gene cluster represents genes which are upregulated in response to all three ligands: AIG, CD40L, and LPS, and includes genes involved in transcription, replication and RNA processing. All three of these ligands induce cellular activation [1,2,15] and proliferation (data not shown), so we would expect these kinds of nuclear genes to be highly expressed in B cells stimulated with all three ligands. The GO term giving the lowest probability in Gene Cluster #6 is "oxidoreductase activity, acting on NADH or NADPH". It has recently been shown that stimulation through CD40 and other TNF family members results in the production of reactive oxygen species through NADPH [16]. Importantly, many of the GO terms giving the low probabilities in a given gene cluster are functionally related, e.g. monovalent inorganic ion transport, ion transport, transporter activity, and cation transport in Gene Cluster #18 (Table 2).

### Experimental validation of CLASSIFI-derived biological predictions

CLASSIFI analysis should be viewed as a hypothesis-generating tool. While the statistical analysis is compelling, predictions that derive from the hypotheses should be verified experimentally. We were particularly interested in the AIG-specific Gene Cluster #18 because these genes represent biological processes that are unique to antigenic stimulation rather than the general activation responses induced by all three ligands. The GO term giving the lowest probability in Gene Cluster #18 is "monovalent inorganic cation transport" (GO:0015672). An examination of the genes annotated with this GO term revealed multiple components of the vacuolar ATPase H+ pump (Table 3). Since stimulation through the BCR induces internalization of the receptor-antigen complex and trafficking through the endocytic system, "monovalent inorganic cation transport" could reflect the acidification of vesicles as they progress from endosomes to lysosomes during endocytosis. This AIG-specific cluster also contained an overrepresentation of genes involved in general "transporter activity" (GO:0005215), which includes genes like Rab9, sorting nexin 5 (Snx5) and N-ethylmaleimide sensitive fusion protein attachment protein alpha (Napa) that are involved in endosome trafficking and vesicle fusion. These observations led us to consider the hypothesis that AIG might induce the expression of various components of the endocytic and vesicle transport pathways in a manner that is independent of its effects on the general metabolic activation and proliferation of B cells, and that this might be part of a B cell-specific antigen processing and presentation function.

**Figure 2:**
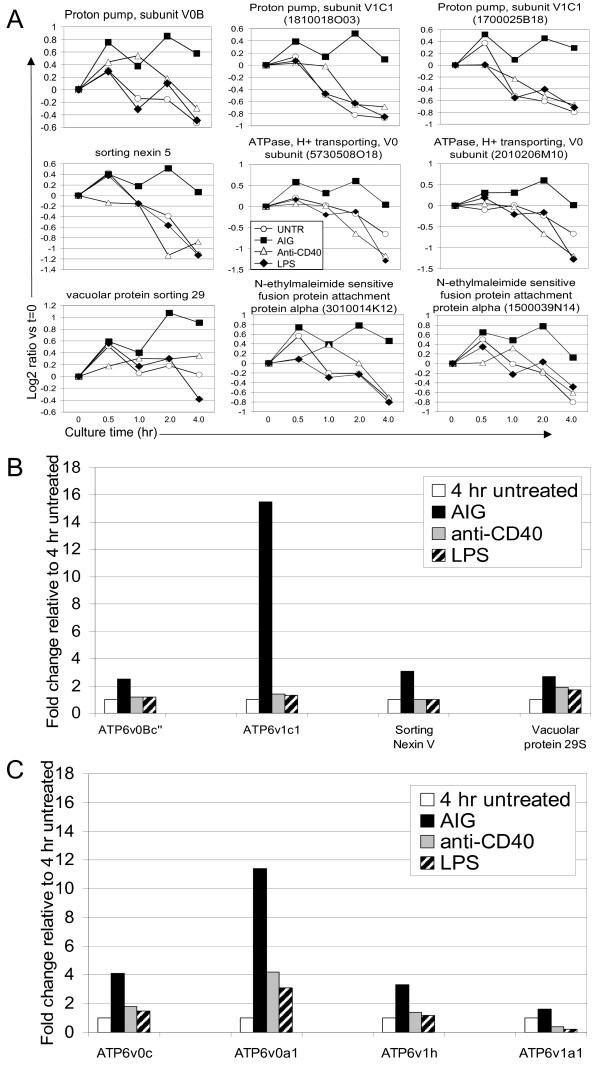
**Expression data from select intracellular transport-related genes**. (A) Microarray results for expression patterns of several genes over a timecourse of 0.5 to 4 hr. The log_2_(Cy5/Cy3) values were compared to 0 hr untreated samples (t = 0). Three examples of genes represented by two different probes (probe Ids in parentheses) are shown in the two right columns. (B) Real-time RT-PCR analysis of four intracellular transport-related genes at 4 hours of stimulation that were included in the microarray data set. Data were normalized to mouse 18S rRNA as a reference and compared to untreated samples. ATP6v0bc" and ATP6v1c1, n = 2. Sorting nexin V and Vacuolar protein 29S, n = 1. (C) Real-time RT-PCR analysis of 4 intracellular transport-related genes at 4 hours of stimulation that were not included in the microarray data set.

To ensure that the genes identified through the CLASSIFI analysis were meaningful, we first sought to verify their differential expression patterns. The microarray expression pattern of nine probes recognizing "transporter activity" genes in unstimulated and ligand-stimulated samples were evaluated (Figure 2A). In every case, expression of these genes was similar in all treatment conditions at 0.5 and 1 hr, but was higher in the AIG-treated samples at 2 hr and 4 hr. Examples in which the same gene is represented by two different probes show closely similar expression patterns, indicating the high quality of this microarray data set. To verify the ligand-specificity of expression, RT-PCR was used to examine the expression of a subset of these genes in independently-generated samples. Again, AIG induced enhanced expression of each of these genes compared to LPS and anti-CD40 (Figure 2B). RT-PCR results consistently confirm microarray expression data in this data set [8].

CLASSIFI analysis is based on the postulate that genes involved in the same biological process are coordinately expressed. Thus, one prediction of this analysis is that other genes involved in the same biological process that were not included in the microarray probe data set would show the same expression pattern. We evaluated 4 such genes that encode ATPase H+ pump complex subunits by RT-PCR. All of these genes demonstrated a preferential upregulation in response to AIG (Figure 2C). These data not only provide experimental validation of the CLASSIFI approach to microarray data analysis, but also further supports the hypothesis that AIG induces vesicle processing and transport as a biological process.

**Figure 3:**
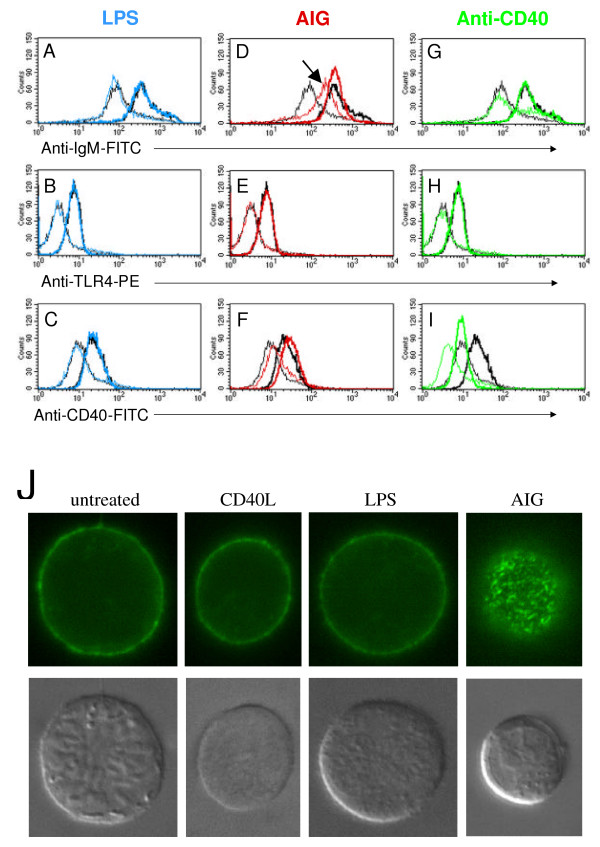
**Internalization of cell surface receptors in WEHI-231**. (A-I) Assay for internalization of BCR, CD40, and TLR4 was performed using WEHI-231 cells. Data from 1 hr stimulations are shown. Line thickness represents treatment of cells following labeling of receptor where thin = acid treated samples and thick = no acid treatment. Black = unstimulated, blue = LPS stimulated, red = AIG stimulated, green = anti-CD40 stimulated. Arrow denotes histogram of acid-treated cells in which labeled BCR is protected from the effects of acid treatment with AIG stimulation. (J) Localization of the BCR by fluorescence microscopy. BCR are labeled with a non-stimulating FITC-conjugated monoclonal anti-IgM antibody (green) and treated for 10 minutes as indicated. DIC images are shown in bottom row.

In order to further test predictions that follow from the CLASSIFI analysis we sought to determine if the process of endocytosis is activated in a ligand/receptor-specific manner. An internalization assay was performed in which the BCR is fluorescently labeled with a non-stimulating anti-IgM-FITC antibody prior to AIG treatment. After allowing time for internalization, the cells are subjected to an acid wash to remove surface-bound label. Label that has been internalized is protected from the acid wash and can therefore be detected using flow cytometry. Using this method, we found that BCR internalization (arrow, Figure 3D) occurs with AIG stimulation, but not CD40L or LPS stimulation in WEHI-231 cells (Figure 3A, G). Substantial internalization was found as early as 15 minutes post-stimulation and continued past 4 hours. Using fluorescence microscopy, internalized BCR can be seen in AIG-treated cells by 10 minutes. No internalization of the BCR is detected with anti-CD40 or LPS treatment (Figure 3J). Ligand-specific internalization of the BCR was also detected in primary B cells (data not shown).

To test whether receptor internalization following AIG stimulation is specific to the BCR and not other cell surface receptors, we performed internalization assays in which TLR4 (Toll-Like Receptor 4 – the LPS receptor) or CD40 was labeled prior to AIG treatment. In both cases, no internalization of the labeled receptor was detected (Figure 3E, F), demonstrating that AIG induces specific internalization of the BCR. (The slight shift in CD40 staining probably results from the upregulation of CD40 in response to AIG treatment.) Finally, to test whether stimulation of TLR4 with LPS or CD40 with anti-CD40 induces TLR4 or CD40 internalization, we fluorescently labeled TLR4 or CD40, then treated cells with the corresponding ligands. In the case of TLR4 stimulation with LPS, no internalization is detected (Figure 3B). Anti-CD40 stimulation induces a rapid receptor shedding phenomenon [17] and so it is difficult to directly compare the results of these samples with the other treatment conditions. However, no evidence for internalization was found (Figure 3I). Thus, AIG induces a ligand-specific, receptor-specific endocytic process that is likely designed to capture specific antigen for processing and presentation.

**Figure 4:**
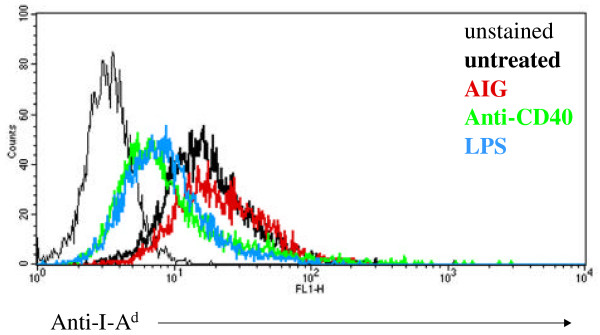
**Class II MHC expression in AIG, anti-CD40, and LPS stimulated WEHI-231**. Flow cytometric analysis of Class II I-A^d ^expression in WEHI-231 in response to overnight stimulation with AIG (red), anti-CD40 (green), and LPS (blue) or media alone (thick black line). Thin black line represents the unstained control.

Finally, MHC class II expression would be expected to be high in B cells undergoing antigen processing and presentation. We found that following overnight stimulation with anti-IgM, CD40L, and LPS, Class II expression on anti-IgM treated cells is sustained, while in CD40L and LPS treated cells Class II expression is significantly downregulated (Figure 4). This ligand-specificity for maintenance of Class II on the surface of anti-IgM-treated cells is interesting because it suggests that B cells constitutively express a high level of Class II on the cell surface in anticipation of antigen presentation, which is then down-regulated in stimulated cells unless they are stimulated through the BCR. This idea that B cells are primed to undergo antigen processing and presentation supports observations in the literature that antigen processing and presentation is accelerated with BCR stimulation. Further, our preliminary data indicate that B cells show enhanced stimulation of antigen-specific T cell clones in response to BCR stimulation (data not shown).

## Discussion

### CLASSIFI analysis

In this paper we report the analysis of gene expression responses of B lymphocytes to a panel of extracellular ligands using CLASSIFI, a statistical approach that uses GO annotation to identify significant co-clustering of related genes, thus linking biological function with gene expression patterns derived from microarray experiments. CLASSIFI circumvents two traditional limitations associated with microarray expression analysis – the effects of experimental and biological noise, and the variable depth of knowledge of individual genes in the probe set. A well-characterized gene has relatively precise annotations that reach deep into the GO hierarchy, while a poorly-characterized gene is only annotated with high level terms. By capturing the entire ontology parentage of each gene, CLASSIFI overcomes limitations associated with variable depth-of-knowledge associated with most gene sets. In addition, the probability calculation used in CLASSIFI is relatively robust to the addition of experimental noise, allowing the use of less stringent data filtering approaches, which reduces the false negative discovery rate without the usual associated increase in false positives. Indeed, increasing the SAM FDR followed by CLASSIFI analysis increase the number of endocytosis-related genes while maintaining similar gene cluster classifications (data not shown).

Our analysis focused on the AIG-specific Gene Cluster #18, which was statistically classified as an intracellular transport-related gene cluster. Inspection of the entire gene list leads to the hypothesis that Gene Cluster #18 classification reflects the broader biological process of BCR-mediated endocytosis, vesicle acidification and trafficking, and antigen processing and presentation. CLASSIFI should be considered a hypothesis-generating tool that requires experimental validation. Several predictions of our hypothesis were confirmed experimentally, including ligand-specific endocytosis induction and MHC class II expression.

It should be noted that although we have used gene ontology annotation as a means of gene classification, any gene description scheme of interest could be used with the CLASSIFI approach to link experimental variables with legacy knowledge through gene expression microarray data.

### Identification of putative components of BCR-mediated antigen capture, processing, and presentation (see Table 4 for reference to this section)

**Figure 5:**
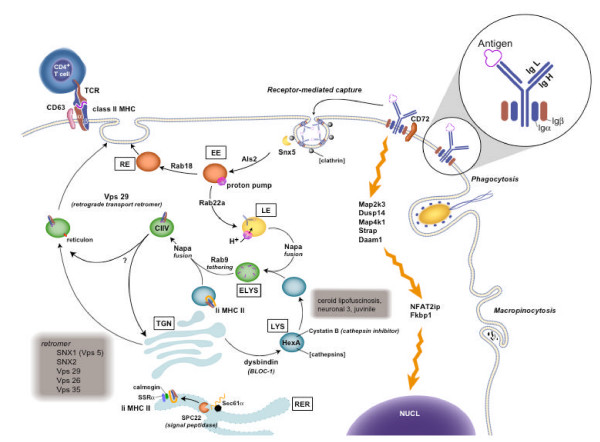
**Gene Cluster #18 contains genes involved in several aspects of vesicle processing, intracellular trafficking, and antigen processing and presentation**. A manual curation of the gene list comprising Gene Cluster #18 resulted in this detailed model of intracellular transport processes in B cells (refer to text for details). EE = early endosome, LE = late endosome, RE = recycling endosome, ELYS = endolysosome, LYS = lysosome, RER = endoplasmic reticulum, TGN = trans-Golgi network, NUCL = nucleus.

Based on the genes responsible for Gene Cluster #18 classification (Table 3), we hypothesized that BCR engagement activates receptor-mediated endocytosis, vesicle acidification, vesicle trafficking, and antigen processing and presentation. We manually curated the AIG-specific cluster gene list and found a total of 38 genes, including those identified using CLASSIFI, that might be involved based on the molecular functions defined in other cell systems and biological processes from the literature (Table 4A). Figure 5 shows a schematic representation of the B cell antigen processing and presentation pathway indicating the putative roles played by these genes.

Several genes known to be involved in signal transduction were found in Gene Cluster #18; some have been demonstrated to play a role in BCR signaling while others are involved in signaling pathways in other cell types and are candidates for new BCR signaling components that stimulate the endocytic process (Table 4A). Activation of protein kinases, including members of the MAP kinase family, in response to BCR engagement is well documented; thus Strap, Map4k1 (Hpk1) and Map2k3 may also play a role in BCR signaling. Indeed, Map4k1 is expressed in hematopoietic cells and is required for activation-induced T cell death following TCR stimulation, and Map2k3 is required for IL-12 production by macrophages and dendritic cells, and antigen-mediated activation of T cells. CD72 has been found to regulate the cellular response to BCR signaling either through the recruitment of the SHP-1 phosphatase thereby dampening BCR signaling, or by cooperating with BCR signaling to prevent apoptosis in immature B cells. The Dusp14 dual specificity phosphatase has been found to associate with the CD28 co-receptor and dampen TCR-mediated signaling in T cells by inactivation of MAP kinases, suggesting that it may regulate BCR signaling in a similar fashion. Daam1 is involved in Wnt signaling in Xenopus embryogenesis. Strap binds to the TGF beta type I receptor and inhibits downstream signaling. Whether these proteins might play a role in regulating BCR signaling remains to be determined. The NF-ATc2 isoform has been found to participate in the BCR-induced apoptosis in B-cell-derived Burkitt's lymphoma cell lines, thus the presence of Nfatc2ip and Fkbp1 suggests that these interacting proteins may also be important in regulating Ca^++^/calmodulin/calcineurin/NFAT signaling in B cells.

A large proportion of genes in Gene Cluster #18 encode proteins with roles in endocytosis, vesicle trafficking, vesicle acidification, molecular processing and protein secretion. In most cases these roles have been defined in other cell systems. Since little is known about the molecular details of these processes in receptor-mediated antigen capture and MHC class II-dependent antigen processing and presentation in B cells, the possibility that Gene Cluster #18 has produced a putative list of proteins that coordinate these activities is intriguing.

Snx5, a member of the sorting nexin family, has been found to interact with clathrin in skeletal muscle. Clathrin has been implicated in facilitating membrane invagination during receptor-mediated endocytosis in B cells. The presence of Snx5 in this cluster suggests that it may help mediate this process. The Als2 protein acts as a guanine nucleotide exchange factor for Rab5 and Rac1, and stimulates early endosome fusion. Rab proteins are small GTPases with homology to Ras that play key roles in vesicle transport: Rab18 has been found to localize near the apical and basolateral plasma membrane in epithelial cells of the kidney and intestine and Rab22a has been found to localize to the plasma membrane and early endosomes. Iqgap1 is a scaffolding protein with multiple protein-protein interaction domains and a GTPase activation protein (Gap) domain that stimulates the hydrolytic activity of Ras family members. Iqgap1 has also been found to localize to areas of membrane ruffling and newly formed vesicles in astrocytoma cells.

Genes encoding homologs to several components of the vesicle-type proton pump were found in Gene Cluster #18, including Atp6v0b, Atp6v1g1, Atp6v1c1, and Atp6v1f and the proton pump accessory protein Atp6ap2. Additional proton pump components were also found to be induced in an AIG-specific manner by RT-PCR (Atp6v0c, Atp6va1, Atp6v1h and Atp6v1a1). Together with the vesicle-specific chloride channel Clcn7, these proteins are likely to play a role in the acidification of the endocytic vesicles in preparation for their fusion with lysosomes to facilitate antigen hydrolysis. Proton pump components can serve as a membrane anchor point for SNARE proteins. Napa appears to mediate the tethering of these vesicles in preparation for fusion. Rab9 also appears to play a role in vesicle fusion since mutations in Rab9 lead to the accumulation of lipid-rich vesicles.

Several proteins involved in lysosome formation and antigen processing were found in Gene Cluster #18. Dysbindin binds to dystobrevins and is a component of the biogenesis of lysosome-related organelles complex 1 in mouse liver, which regulates trafficking to lysosomal organelles. Mutations in the Cln3 gene leads to a lysosomal storage disorder associated with the accumulation of lipopigment-laden vesicles. Hexosaminidase A is a pH-sensitive hydrolase. A variety of cathepsins are involved in MHC Class II antigen processing and presentation. None of the probes for these genes passed our data filtering process, however the cathepsin inhibitor Cystatin B was found in Gene Cluster #18, suggesting that it might play a role in regulating cleavage site selection by modifying the relative activities of proteases involved in antigen processing and peptide loading. Hspbp1 may facilitate antigen processing by regulating the chaperone function of Hsp70, which has been associated with the presentation of myelin basic protein though MHC class II in multiple sclerosis.

Several genes involved in MHC class II expression were found in the AIG-specific gene cluster. Atf1 is a transcription factor that activates the promoter of the class II *trans*-activator (CIITA). Translocation of nascent MHC class II peptide chains into the lumen of the endoplasmic reticulum involves the function of the Sec61 translocation complex, the signal sequence receptor Ssr1 (a.k.a. TRAP alpha) and the signal peptidase SPC22. Calmegin (Clgn) is a Ca^++ ^binding chaperone protein with significant homology to calreticulin and calnexin, an ER chaperone protein that regulates the assembly of MHC class II with the Ii invariant chain.

Vesicles containing peptide-loaded MHC class II are transported to the plasma membrane for fusion and exposure on the cell surface, perhaps by traveling back through the TGN into the secretory pathway. The Vsp29 protein has been found to facilitate this kind of retrograde TGN transport in yeast. In neurons, reticulon 3 is associated with synaptophysin in tubulovesicular structures and may play a role in the process of vesicle secretion. CD63, an MHC class II co-receptor, may play a role in regulating MHC receptor compartmentalization.

38 genes found in Gene Cluster #18 encode proteins that have some connection with signaling, antigen capture, vesicle transport, vesicle acidification or MHC class II expression, suggesting that activation of B cells through the BCR is inducing the expression of genes involved in MHC class II antigen processing and presentation. The activation of class II presentation by antigen receptor engagement in B cells has been described extensively. The findings reported here are novel in that microarray results combined with CLASSIFI analysis have provided a list of genes that may be involved in this important immunological process.

Finally, it is intriguing to note the presence of genes involved in the regulation of apoptosis and autoimmunity (Table 4C and 4D) in Gene Cluster #18, given the known function of the BCR in negative selection and the induction of autoimmunity.

### Transcriptional positive feedback

The changes in mRNA levels for these genes involved in vesicle trafficking might be necessary to stimulate this biological process in AIG-stimulated B cells. However, the kinetics of the mRNA changes compared with the rapid induction of endocytosis suggests that this may not be the case. Elevated mRNA levels for these genes was found at 2 and 4 hr, but not at 0.5 or 1 hr post-stimulation. However, BCR endocytosis was maximally stimulated by 15 minutes. The rapid induction of endocytosis suggests that at least the initiation of this process is activated through post-transcriptional mechanisms. This finding suggests that the AIG-specific transcriptional reprogramming observed in response to BCR stimulation may reflect positive feedback regulation in which expression of proteins that have already been activated is increased either as a means to amplify or sustain the process. Perhaps components of this important biological process are present at low levels in resting cells, and levels increase in response to appropriate environmental cues. This kind of regulatory process might allow cells to respond rapidly to a broad set of variables in their changing environment while conserving energy and materials while in a resting state.

## Conclusion

In this paper, we describe a bioinformatics analysis of a B cell microarray dataset using CLASSIFI to determine the biological significance of microarray gene clusters defined by ligand-specific B cell responses. Importantly, results from laboratory experimentation support the hypotheses derived from this microarray data mining exercise. This work is a model for immunologists and other biologists for utilizing the growing field of bioinformatics for microarray data mining, hypothesis generation and hypothesis testing. Furthermore, the results from this analysis provide a foundation for a more detailed understanding of the B cell antigen processing and presentation pathway.

## Methods

### Primary B cell isolation and culture

Isolation of primary splenic murine B cells was performed as described in the Alliance for Cellular Signaling (AfCS)/Nature Signaling Gateway website [18]. Briefly, splenocytes from 6–10 week old C57BL/6 mice were subjected to a magnetic bead negative selection procedure (Miltenyi Biotech, Auburn, CA) to remove CD43+ and Mac-1+ cells, resulting in an enriched population of resting B cells that was an average of 96% B220+, as determined by flow cytometry [19]. Purified B cells were cultured in supplemented Iscove's Modified Dulbecco's Medium [20] with 100 U/ml penicillin and 100 μg/ml streptomycin (Invitrogen, Carlsbad, CA) at 37°C under 5% CO_2_. For microarray and internalization experiments, cells were treated for 0.5 hr, 1 hr, 2 hr, and 4 hr with media alone, or with mu chain specific Goat anti-mouse immunoglobulin, (AIG) (Jackson Immunoresearch) at 45 μg/ml, hamster anti-mouse CD40 monoclonal antibody, clone HM40-3 (BD Pharmingen) at 9.73 μg/ml, or lipopolysaccharide (LPS) (Sigma-Aldrich) at 40 μg/ml. For real-time PCR experiments, cells were treated with anti-IgM at 20 μg/ml or anti-CD40 at 625 ng/ml.

### Microarray analysis and clustering

Microarray-related experimental protocols can be found at the AfCS/Nature website at [21]; protocol IDs are: PP00000009-RNA extraction, PP00000019-sample preparation and hybridization. Briefly, RNA samples isolated from B cells treated with a panel of 32 ligands were compared with untreated samples using a microarray chip containing 15,494 cDNA probes printed on 15,832 spots representing 10,615 unique MGI gene matches (as of 12/31/03). 38% of the probes have not been assigned a gene name, 96% come from the RIKEN FANTOM collection, 3% from the Minoro Ko National Institute of Aging collection, and the rest from the Research Genetics and Genome Systems collections. RNA samples were used to generate Cy5- and Cy3-labeled cDNA targets (from sample RNAs and RBC-depleted total splenocyte RNA, respectively) and were hybridized together. All samples were run in triplicate except for 1 hr and 4 hr untreated controls, which were run in quadruplicate. Arrays were scanned using the Agilent Scanner G2505A (Agilent Technologies, Palo Alto, CA). Image files were analyzed using the Agilent G2566AA Feature Extraction software version A.6.1.1. The raw data from these experiments is available at [22]. Spot features on each array were filtered to remove those values that were saturated, non-uniform, or below background. Statistical filtering was accomplished using Significance Analysis of Microarrays (SAM) [23]. Features found by SAM to be differentially expressed between samples and time-matched untreated controls at a false discovery rate (FDR) of 1% were included for further analysis. Input for SAM were background-subtracted, dye bias- and interarray variance-normalized Cy5 fluorescence intensity values, which represent expression level of array features. Only features with more than two replicates were used in the SAM analysis. 100 random permutations were done for each comparison of treated to time-matched control samples. Values of +1, -1, or 0 were given to genes that were found by SAM to be significantly upregulated, downregulated, or unchanged (respectively) compared to time-matched untreated controls. These values were used to categorically group genes together based on their expression response patterns.

### CLASSIFI analysis

*Cl*uster *Assi*gnment for Biological *I*nference (CLASSIFI) was developed as a method for statistical evaluation of Gene Ontology™ (GO) term co-clustering (Figure 1A). CLASSIFI is predicated on the postulate that genes involved in the same biological process are coordinately expressed; examples of coordinate expression of interacting proteins in eukaryotes has been described [24-27]. Following data transformation, filtering, normalization, standard expression clustering approaches and gene cluster membership assignment, the following steps are performed in the CLASSIFI analysis: 1) remove duplicate probe IDs, 2) extract the primary GO annotations for each gene from a probe database, 3) capture the full GO ancestry for each primary GO annotation from the Gene Ontology™ database, and 4) calculate the solution for the cumulative hypergeometric distribution equation for every GO term in every gene cluster:

P=1−∑i=0n−1(fi)(g−fc−i)(gc)
 MathType@MTEF@5@5@+=feaafiart1ev1aaatCvAUfKttLearuWrP9MDH5MBPbIqV92AaeXatLxBI9gBaebbnrfifHhDYfgasaacH8akY=wiFfYdH8Gipec8Eeeu0xXdbba9frFj0=OqFfea0dXdd9vqai=hGuQ8kuc9pgc9s8qqaq=dirpe0xb9q8qiLsFr0=vr0=vr0dc8meaabaqaciaacaGaaeqabaqabeGadaaakeaacqWGqbaucqGH9aqpcqaIXaqmcqGHsisldaaeWbqaamaalaaabaWaaeWaaeaafaqabeGabaaabaGaemOzaygabaGaemyAaKgaaaGaayjkaiaawMcaamaabmaabaqbaeqabiqaaaqaaiabdEgaNjabgkHiTiabdAgaMbqaaiabdogaJjabgkHiTiabdMgaPbaaaiaawIcacaGLPaaaaeaadaqadaqaauaabeqaceaaaeaacqWGNbWzaeaacqWGJbWyaaaacaGLOaGaayzkaaaaaaWcbaGaemyAaKMaeyypa0JaeGimaadabaGaemOBa4MaeyOeI0IaeGymaedaniabggHiLdaaaa@4AE3@

where *g *= number of probes in the data set, *c *= number of probes in the gene cluster, *f *= number of probes with a given ontology in the data set, *n *= number of probes with a given ontology in the gene cluster. The hypergeometric distribution calculates the probability (*P*) that genes with a particular GO term would co-cluster by chance given the proportion of genes annotated with this GO term in the entire data set. A web interface for use of the CLASSIFI method with data derived from cDNA, oligonucleotide and Affymetrix microarrays, along with detailed information about CLASSIFI input and output files can be found at the CLASSIFI website [28].

### Quantitative real-time reverse-transcription-polymerase chain reaction

Total RNA was extracted as for the microarray experiments. 1 μg of total RNA was treated with DNaseI (Invitrogen), then reverse transcribed at 42°C for one hour in a 20 μl volume containing 1 μl MMLV reverse transcriptase (Invitrogen), 4 μl 5X buffer (Invitrogen), 0.5 mM dNTPs (Invitrogen), and 5 ng/μl pd(N)_6 _(Amersham). Following inactivation at 70°C for 20 min, PCR reactions were set up in a 20 μl volume using Sybr Green Master Maker (Applied Biosystems). Thermal cycling began with a denaturation step of 10 minutes at 95°C, followed by 40 cycles of 95°C for 15 seconds (denaturation) and 60°C for 1 minute (annealing and extension). PCR reactions were performed in the ABI Prism 7700 Sequence Detector (PE Biosystems, Foster City, CA), and data collected and analyzed with the Sequence Detector software (PE Biosystems). Standard curves were generated using RNA isolated from RBC-depleted mouse splenocytes. RT-PCR of mouse 18S rRNA levels was used for normalization. Data from treated samples were compared to untreated samples, giving values representing the fold change in gene expression relative to untreated samples. PCR primers were designed using Primer3 software [29]: ATP6v0Bc" forward (f) 5'gaaccccagcctctttgtaa3', reverse (r) 5'cccatcttcactctggaggt3'; ATP6v1c1 (f) 5'tgcttgccaaagaggtaaca3', (r) 5'tcgctgcatgtagtttctcc3'; sorting nexin V (f) 5'gggagaaggggaaggatcta3', (r) 5'catgggtggacacagtcttc3'; vacuolar protein 29S (f) 5'ctgcagaggcagtttgatgt3', (r) 5'ggcagaacctgggttaatgt3'; ATP6v0c (f) 5'atgtcagtcatgaggccaga3', (r) 5'agcgataagtactgccacca3'; ATP6v0a1 (f) 5'tccacccagtctgtaggtga3'. (r) 5'atcatgatcagggtgcagaa3'; ATP6v1h (f) 5'gatgctgctgtcccaactaa3', (r) 5'agaaatcatctgcccctgaa3'; ATP6v1a1 (f) 5'gaattatgatgcgtccgatg3', (r) 5'cgcctgggatagcagtagtt3'. Mouse 18S PCR primers sequences are from [30]: (f) 5'gtaacccgttgaaccccatt3', (r) 5'ccatccaatcggtagtagcg3'.

### Internalization assays and flow cytometry

1 × 10^6 ^WEHI-231 cells (a mouse B cell lymphoma line) were pre-stained for 10 min at 4°C with monoclonal antibodies to cell surface receptors: Rat anti-IgM-FITC clone R6-60.2 or clone II/40 (BD Pharmingen), Rat anti-CD40-FITC clone 3/23 (BD Pharmingen), or Rat anti-TLR4/MD2-PE clone MTS510 (eBiosciences). Following 2 washes in PBS/2% FBS/2 mM EDTA, cells were cultured and stimulated using the aforementioned ligand concentrations in RPMI supplemented with 10% FBS (Hyclone), 100 U/ml penicillin, 100 μg/ml streptomycin (Invitrogen, Carlsbad, CA), sodium pyruvate, HEPES, and 2-ME (Invitrogen). Following incubation at 37°C with 5% CO_2 _for various time periods, cells were harvested and incubated at 4°C for 5 minutes in 0.2 M Acetic acid/0.5 M NaCl to strip off staining antibodies [31], or in PBS/2% FBS/2 mM EDTA (control). Following 2 washes in 0.2 M Acetic acid/0.5 M NaCl or PBS/2% FBS/2 mM EDTA, respectively, samples were fixed in 1% paraformaldehyde. Data were acquired using a FACSCalibur™ flow cytometer and analyzed using CellQuest™ software (Becton Dickinson Immunocytometry Systems).

MHC class II expression was measured by flow cytometric analysis using FITC-conjugated antibody against I-A^d ^clone 39-10-8 (BD Pharmingen).

### BCR localization by immunofluorescence microscopy

1 × 10^6 ^WEHI-231 cells were harvested and resuspended in 100 μl PBS containing 2% FBS. 2 μl of rat anti-IgM-FITC clone R6-60.2 antibody was added, and cells incubated on ice for 10 minutes to allow BCR staining. R6-60.2 was determined to be non-stimulating because it did not flux calcium or induce detectable tyrosine phosphorylation in these cells (data not shown). Following one wash in PBS/2%FBS, cells were resuspended in 100 ul phenol-red free growth medium (Invitrogen) and kept on ice prior to stimulation. Cells were stimulated by addition of ligands at the following concentrations: AIG, 20 μg/ml; HM40-3, 9.73 μg/ml; or LPS, 40 μg/ml. Images were captured at room temperature immediately following ligand stimulation for up to 10 min. Using the Zeiss Axioplan 2 upright fluorescence microscope (Carl Zeiss, Inc, Thornwood, NY), images were captured with the 60X objective under oil immersion using the OpenLab™ software, version 4.0.1 (Improvision, Boston, MA). 2 images were captured for each field of view: one for detection of FITC fluorescence, and one for differential interference contrast (DIC) imaging of cells. Images were processed using OpenLab™.

## Abbreviations

Ligand abbreviations:

2MA - 2-Methyl-thio-ATP

AIG - Antigen (Anti-Ig)

BAF - BAFF (B-cell activating factor)

BLC - BLC (B-lymphocyte chemoattractant)

BOM - Bombesin

40L - CD40 ligand

CGS - CGS-21680 hydrochloride (2-p-[2-Carboxyethyl]phenethylamino-5'-N-ethylcarboxamidoadenosine)

CPG - CpG-Containing Oligonucleotide

DIM - Dimaprit

ELC - ELC (Epstein Barr Virus-induced molecule-1 Ligand Chemokine)

FML - fMLP (formyl-Met-Leu-Phe)

GRH - Growth hormone-releasing hormone

IGF - Insulin-like growth factor 1

IFB - Interferon-beta

IFG - Interferon-gamma

I10 - Interleukin 10

I04 - Interleukin 4

LPS - Lipopolysaccharide

LB4 - Leukotriene B4 (LTB4)

LPA - Lysophosphatidic acid

M3A - MIP3-alpha (Macrophage inflammatory protein-3)

NEB - Neurokinin B

NPY - Neuropeptide Y

NGF - NGF (Nerve Growth Factor)

PAF - Platelet activating factor

PGE - Prostaglandin E2

SDF - SDF1 alpha (Stromal cell derived factor-1)

SLC - SLC (Secondary lymphoid-organ chemokine)

S1P - Sphingosine-1-phosphate

TER - Terbutaline

TNF - Tumor necrosis factor-alpha

TGF - Transforming growth factor-beta 1

Other Abbreviations:

BCR - B cell receptor

CLASSIFI - Cluster Assignment for Biological Inference

GO - Gene Ontology

SAM - Significance Analysis of Microarrays

TLR4 - Toll-Like Receptor 4

## Authors' contributions

JL carried out the microarray clustering, CLASSIFI analysis and experimental validation, and drafted the manuscript. RSS and DM participated in the microarray analysis and CLASSIFI design and implementation. ER and RCH participated in experimental validation. JC and PY participated in implementing the CLASSIFI algorithm as a web-based application. BS and SS participated in the CLASSIFI design and implementation. SC participated in the microarray analysis. RHS conceived of the study and the CLASSIFI algorithm, participated in its design, coordinated the study and helped to draft the manuscript. All authors participated in critical review of the manuscript and give final approval for the submitted manuscript.

## Supplementary Material

Additional File 1 **CLASSIFI input file**. The CLASSIFI input file was generated following microarray data clustering. It lists probe IDs, Gene names, and Cluster IDs resulting from categorical clustering of processed data from B cells stimulated with anti-CD40, LPS, and AIG. This input file was uploaded at the CLASSIFI website [28], which provides a web interface for the use of CLASSIFI. Click here for file

Additional File 2 **CLASSIFI output file**. Complete output file resulting from CLASSIFI analysis of the data set. Clusterid = gene clusters resulting from categorical clustering of processed data from B cells stimulated with anti-CD40, LPS, and AIG. GO id = a unique Gene Ontology identifier that corresponds to a GO term, which is used to describe gene products based on molecular function, biological process, or cellular component. g = number of probes in the data set, f = number of probes with associated GO id in the data set, c = number of probes in the gene cluster, n = number of probes with associated GO id in the gene cluster. Expt = the expected number of occurrences of a given GO id in a given cluster of size (n) based on a random distribution. Prob = the probability that the GO id co-cluster pattern has occurred by chance. Click here for file

Additional File 3 **CLASSIFI GO file**. Complete list of all GO ids (representing the entire GO ancestry) associated with each probe ID. Click here for file

## Figures and Tables

**Table 1:** Clustering and CLASSIFI results for data from 3 ligands

GO ID	g	f	c	n	expt	prob	GO type	GO term	Cluster ID	anti-CD40	LPS	AIG
GO:0005634	2490	254	380	64	38.76	8.71E-06	CC	Nucleus	1	1	1	1
GO:0009058	2490	141	180	25	10.19	1.36E-05	BP	biosynthesis	2	1	1	
GO:0008372	2490	1409	1	1	0.566	5.66E-01	CC	CC unknown	3	1	-1	-1
GO:0046072	2490	2	160	2	0.129	4.10E-03	BP	dTDP metabolism	4	1		1
GO:0009605	2490	30	3	2	0.036	4.18E-04	BP	response to external stimulus	5	1		-1
GO:0016655	2490	14	331	11	1.861	4.94E-08	MF	oxidoreductase activity, acting on NADH	6	1		
GO:0005773	2490	12	11	4	0.053	1.00E-07	CC	Vacuole	7	-1	-1	1
GO:0003779	2490	15	277	9	1.669	6.30E-06	MF	actin binding	8	-1	-1	-1
GO:0016758	2490	6	245	4	0.59	1.17E-03	MF	Transferase activity, transferring hexosyl groups	9	-1	-1	
GO:0006417	2490	2	4	1	0.003	3.21E-03	BP	regulation of protein biosynthesis	10	-1		1
GO:0008372	2490	1409	41	32	23.2	3.30E-03	CC	CC unknown	11	-1		-1
GO:0008047	2490	10	160	3	0.643	2.23E-02	MF	enzyme activator activity	12	-1		
GO:0006397	2490	19	56	4	0.427	6.92E-04	BP	mRNA processing	13		1	1
GO:0005576	2490	156	183	33	11.47	7.28E-09	CC	extracellular	14		1	
GO:0046916	2490	1	4	1	0.002	1.61E-03	BP	transition metal ion homeostasis	15		-1	1
GO:0003931	2490	3	38	2	0.046	6.74E-04	MF	Rho small monomeric GTPase activity	16		-1	-1
GO:0004032	2490	3	188	3	0.227	4.24E-04	MF	aldehyde reductase activity	17		-1	
GO:0015672	2490	10	191	7	0.767	1.38E-06	BP	monovalent inorganic cation transport	18			1
GO:0016892	2490	3	50	2	0.06	1.17E-03	MF	endoribonuclease activity, producing other than	19			-1

Gene clusters (Cluster ID 1–19) resulting from categorical clustering of processed data from B cells stimulated with anti-CD40, LPS, and AIG. "1" = upregulated, "-1" = downregulated, and blank = no significant change. Following CLASSIFI analysis, the GO term giving rise to the lowest probability in each gene cluster is indicated. GO ID = a unique Gene Ontology identifier assigned to a GO term that corresponds to a defined molecular function (MF), biological process (BP), or cellular component (CC). g = number of probes in the data set, f = number of probes with associated GO ID in the data set, c = number of probes in the gene cluster, n = number of probes with associated GO ID in the gene cluster. Expt = the expected number of occurrences of a given GO ID in a given cluster of size (n) based on a random distribution. Prob = the probability that the GO ID co-cluster pattern has occurred by chance.

**Table 2:** Excerpt of CLASSIFI results for six gene clusters giving significant probabilities

GO ID	f	n	prob	GO type	GO term	Cluster ID
GO:0005634	254	64	8.71E-06	CC	nucleus	1
GO:0005635	13	9	1.66E-05	CC	nuclear membrane	1
GO:0006412	94	30	2.75E-05	BP	protein biosynthesis	1
GO:0009059	94	30	2.75E-05	BP	macromolecule biosynthesis	1
GO:0003743	20	10	2.68E-04	MF	translation initiation factor activity	1
GO:0016655	14	11	4.94E-08	MF	oxidoreductase activity, acting on NADH or NADPH, quinone or similar compound as acceptor	6
GO:0008137	14	11	4.94E-08	MF	NADH dehydrogenase (ubiquinone) activity	6
GO:0015081	14	11	4.94E-08	MF	sodium ion transporter activity	6
GO:0016651	16	11	4.61E-07	MF	oxidoreductase activity, acting on NADH or NADPH	6
GO:0046873	16	11	4.61E-07	MF	metal ion transporter activity	6
GO:0005773	12	4	1.00E-07	CC	vacuole	7
GO:0016787	163	6	2.52E-05	MF	hydrolase activity	7
GO:0005624	15	3	2.84E-05	CC	membrane fraction	7
GO:0000267	18	3	5.05E-05	CC	cell fraction	7
GO:0015991	3	2	5.31E-05	BP	ATP hydrolysis coupled proton transport	7
GO:0003779	15	9	6.30E-06	MF	actin binding	8
GO:0008092	22	9	3.08E-04	MF	cytoskeletal protein binding	8
GO:0003785	6	4	1.88E-03	MF	actin monomer binding	8
GO:0006357	10	5	2.58E-03	BP	regulation of transcription from Pol II promoter	8
GO:0006355	79	17	4.85E-03	BP	regulation of transcription, DNA-dependent	8
GO:0005576	156	33	7.28E-09	CC	extracellular	14
GO:0005578	5	4	1.33E-04	CC	extracellular matrix	14
GO:0004871	50	11	7.39E-04	MF	signal transducer activity	14
GO:0004888	7	4	8.29E-04	MF	transmembrane receptor activity	14
GO:0007166	25	7	1.59E-03	BP	cell surface receptor linked signal transduction	14
GO:0015672	10	7	1.38E-06	BP	monovalent inorganic cation transport	18
GO:0006811	23	10	2.65E-06	BP	ion transport	18
GO:0005215	122	24	9.10E-06	MF	transporter activity	18
GO:0006812	18	8	2.33E-05	BP	cation transport	18
GO:0006754	4	4	3.36E-05	BP	ATP biosynthesis	18

The five GO IDs giving the lowest probability are listed for each of six gene clusters giving significant probabilities in Table 1. GO ID = a unique Gene Ontology identifier that corresponds to a defined molecular function, biological process, or cellular component (GO name). f = number of probes with the associated GO ID in the data set, n = number of probes with the associated GO ID in the gene cluster. prob = the probability that the GO ID co-cluster pattern has occurred by chance.

**Table 3:** Genes annotated with transport-related GO terms from Gene Cluster #18

GO ID	Probe ID	Gene Name
GO:0015672	C530010I21	ATPase, H+ transporting, V0 subunit B
GO:0015672	2310069H14	ATPase, H+ transporting, V1 subunit G isoform 1
GO:0015672	5730403E06	SIMILAR TO ATPASE, H+ TRANSPORTING, LYSOSOMAL
GO:0006811	2810002O05	sideroflexin 1
GO:0006811	1810048H22	arsA (bacterial) arsenite transporter, ATP-binding, homolog 1
GO:0006811	2310021D14	chloride channel 7
GO:0005215	1810011E08	MICROSOMAL SIGNAL PEPTIDASE 23 KDA SUBUNIT (EC 3.4.-.-)
GO:0005215	2810433C04	translocase of inner mitochondrial membrane 9 homolog (yeast)
GO:0005215	1500039N14	N-ethylmaleimide sensitive fusion protein attachment protein alpha
GO:0005215	2210039G11	fatty acid binding protein 5, epidermal
GO:0005215	5430437K12	zinc finger protein 295
GO:0005215	1810057M21	thioredoxin reductase 1
GO:0005215	2310004L13	translocase of inner mitochondrial membrane 9 homolog (yeast)
GO:0005215	2610025H03	fatty acid binding protein 5, epidermal
GO:0005215	5430413F24	RAB9, member RAS oncogene family
GO:0005215	5830417J06	24-dehydrocholesterol reductase
GO:0005215	0910001N05	sorting nexin 5
GO:0005215	2410046L22	FK506 binding protein 1a
GO:0005215	2010015D08	vacuolar protein sorting 29 (S. pombe)
GO:0005215	3010014K12	N-ethylmaleimide sensitive fusion protein attachment protein alpha

List of characterized genes detected using specific cDNA probes (Probe ID) found in Gene Cluster #18 that are associated with GO terms "monovalent inorganic ion transport" (GO:0015672), "ion transport" (GO:0006811), and "transporter activity" (GO:0005215). N.B. GO:0015672 is a child of GO:0006811, which is a child of GO:0005215 in the GO hierarchy.

**Table 4:** Manual curation of Gene Cluster #18 genes

	Protein description	Gene Sym Symbol	LocusLink ID	Function	Reference
A	CD72	Cd72	12517	BCR coreceptor	32–34 35
	dual specificity phosphatase 14	Dusp14	56405	lymphocyte signaling	36
	mitogen activated protein kinase kinase kinase kinase 1	Map4k1	26411	lymphocyte signaling	37–39
	mitogen activated protein kinase kinase 3	Map2k3	26397	lymphocyte signaling	40, 41
	serine/threonine kinase receptor associated protein	Strap	20901	TGFbeta signaling	42
	dishevelled associated activator of morphogenesis 1	Daam1	208846	Wnt signaling	43
	FK506 binding protein 1a	Fkbp1a	14225	BCR signaling/apoptosis	44–46
	nuclear factor of activated T-cells, cytoplasmic 2 interacting protein	Nfatc2ip	18020	BCR signaling/apoptosis	44–46
	sorting nexin 5	Snx5	69178	endocytosis	47, 48
	IQ motif containing GTPase activating protein 1	Iqgap1	29875	vesicle formation	49, 50
	amyotrophic lateral sclerosis 2 (juvenile) homolog (human)	Als2	74018	endosome fusion/Rab GEF	51, 52
	RAB18, RAS family	Rab18	19330	endocytosis/vesicle recycling	53–56 56
	RAB22, RAS family	Rab22a	19334	early endosome trafficking	56, 57
	Proton pump, subunit V0B (21 kDa)	Atp6v0b	114143	vesicle acidification	58–60
	Proton pump, subunit V1G1 (13 kDa)	Atp6v1g1	66290	vesicle acidification	58–60
	Proton pump, subunit V1C1 (42 kDa)	Atp6v1c1	66335	vesicle acidification	58–60
	similar to Proton pump, subunit V1F		76610	vesicle acidification	58–60
	Proton pump accessory protein 2	Atp6ap2	70495	vesicle acidification	61, 62
	Chloride channel 7	Clcn7	26373	vesicle pH regulation	63, 64
	dystrobrevin binding protein 1 (dysbindin)	Dtnbp1	94245	lysosome formation	65
	cystatin B	Cstb	13014	cathepsin protease inhibitor	66–68
	hexosaminidase A	Hexa	15211	hydrolase	69–71
	ceroid lipofuscinosis, neuronal 3, juvenile (Batten, Spielmeyer-Vogt disease)	Cln3	12752	lysosome function	72
	HSP70 binding protein	Hspbp1	66245	Ag processing/loading	73
	similar to HSPC135		66067	chaperone (?), GTPase (?)	
	HSPC245 homolog			chaperone (?)	
	N-ethylmaleimide sensitive fusion protein attachment protein alpha	Napa	108124	vesicle fusion	74, 75
	RAB9, RAS family	Rab9	56382	vesicle tethering	76, 77
	activating transcription factor 1	Atf1	11908	MHC expression	78
	Sec61 alpha	Sec61a1	53421	ER translocation	79
	signal sequence receptor, alpha	Ssr1	107513	MHCII chaperone	80
	calmegin	Clgn	12745	MHC chaperone	81, 82
	similar to signal peptidase SPC22/23		76687	secretory protein processing	83
	vacuolar protein sorting 29	Vps29	56433	retrograde vesicle transport	84, 85
	reticulon 3	Rtn3	20168	exocytosis	86, 87
	tubulin, alpha 6	Tuba6	22146	cytoskeletal trafficking	88, 89
	CD63	Cd63	12512	MHCII coreceptor	90–92
	source of immunodominant MHC-associated peptides	Simp	68292	MHC peptide	93
					
B	24-dehydrocholesterol reductase	Dhcr24	74754	Cholesterol biosynthesis	94
	fatty acid binding protein 5, epidermal	Fabp5	16592	Fatty acid intracellular transport	95
	phosphatidylethanolamine binding protein	Pbp	23980	Membrane lipid transport	96
	prostaglandin D2 synthase 2, hematopoietic	Ptgds2	54486		
	peroxisome biogenesis factor 1	Pex1	71382	Peroxisomal protein tranport	97
					
C	beclin 1 (coiled-coil, myosin-like BCL2-interacting protein)	Becn1	56208	Tumor suppression	98, 99
	caspase 4, apoptosis-related cysteine protease	Casp4	12363	Apoptosis	100–102
	Cd27 binding protein (Hindu God of destruction)	Siva	30954	Apoptosis	103, 104
					
D	polymyositis/scleroderma autoantigen 2	Pmscl2	50912	RNA processing autoantigen	105
	Sjogren's syndrome/scleroderma autoantigen 1 homolog (human)	Sssca1	56390	centromeric autoantigen	106
	amyotrophic lateral sclerosis 2 (juvenile) homolog (human)	Als2	74018	endosome fusion/Rab GEF	51, 52

Genes found in Gene Cluster #18 with functions related to (A) intracellular trafficking, BCR signaling, vesicle processing and function, and antigen processing and presentation, (C) apoptosis, and (D) B cell-related autoimmune disease. (B) Genes annotated with "transporter" activity whose roles are not yet known to be associated with intracellular trafficking.
